# An update on the role of thioredoxin-interacting protein in diabetic kidney disease: A mini review

**DOI:** 10.3389/fmed.2023.1153805

**Published:** 2023-04-18

**Authors:** Hong Sun, Rong Sun, Yulin Hua, Qianyi Lu, Xinyu Shao

**Affiliations:** ^1^Department of Endocrinology and Metabolism, Dushu Lake Hospital Affiliated to Soochow University, Medical Center of Soochow University, Suzhou, Jiangsu, China; ^2^The First Clinical Medical College, Soochow University, Suzhou, Jiangsu, China; ^3^Department of Ophthalmology, The First Affiliated Hospital of Soochow University, Suzhou, Jiangsu, China; ^4^Department of Ophthalmology, Changshu No. 1 People’s Hospital, Suzhou, Jiangsu, China

**Keywords:** diabetic kidney disease, thioredoxin-interacting protein, reactive oxygen species, autophagy, lipid

## Abstract

Thioredoxin-interacting protein (TXNIP) was first isolated from Vitamin D3-exposed HL60 cells. TXNIP is the main redox-regulating factor in various organs and tissues. We begin with an overview of the TXNIP gene and protein information, followed by a summary of studies that have shown its expression in human kidneys. Then, we highlight our current understanding of the effect of TXNIP on diabetic kidney disease (DKD) to improve our understanding of the biological roles and signal transduction of TXNIP in DKD. Based on the recent review, the modulation of TXNIP may be considered as a new target in the management of DKD.

## Introduction

Diabetic kidney disease (DKD) is the main diabetes-related complication and a major factor resulting in end-stage renal disease (ESRD). It imposes a substantial financial burden and greatly affects the lives of the patients and their families. Hyperglycemia is the primary factor but not the only etiological factor inducing DKD. The initiation and maintenance of intrarenal pathogenetic pathways due to an increase in glucose concentrations are aided by a variety of processes such as dyslipidemia, oxidative stress, inflammation, vascular perfusion changes, and excessive activation of the renin-angiotensin-aldosterone system (RAAS) (1). Medical studies have shown that DKD has the main clinical symptoms of hypertension and proteinuria, along with gradually declining kidney function (2). Typical morphological alterations in DKD involve mesangial cell proliferation, podocyte loss, glomerular hypertrophy, glomerular sclerosis, tubular injury, interstitial fibrosis, and thickening of the basement membrane. Overall, DKD shows high heterogeneity in the clinical symptoms, histopathological changes, and progression rate; thus, making it difficult to formulate efficient treatment methods.

The thioredoxin-interacting protein (TXNIP) has received a lot of attention in recent years because it has a wide range of activities and influences many metabolic processes. Many studies have suggested that TXNIP plays an important role in the onset and progression of diabetes, and TXNIP epigenetic regulation is linked to diabetes prevalence (3, 4). Studies on mice have suggested that TXNIP deficiency can enhance glucose homeostasis while ameliorating insulin resistance (5). Furthermore, anti-diabetic medications, such as glucagon-like peptide-1 (GLP-1) and metformin, can enhance glucose homeostasis by regulating TXNIP (6, 7). Moreover, several experts have elucidated the mechanism of TXNIP in the progression of diabetes and its role in the treatment of diabetes (8). Additionally, TXNIP expression increases in the kidneys of diabetic patients and model rodents and is closely related to changes in kidney morphology and function (9–14). However, the effect of TXNIP on the pathogenic mechanism of DKD is rarely generalized. In this study, we summarized the information regarding TXNIP biology and emphasized its effect on DKD to facilitate further studies. The studies on TXNIP and DKD have been summarized in Table 1.

**Table 1:** Effect of TXNIP for DKD in experimental studies.

Year	First author	Diabetic model	Results				*In vitro*	Human renal biopsy	Interpretation	Ref.
			Metabolic parameter	Kidney function	Renal pathological changes	Molecular signaling pathway				
2009	Andrew Advani	mRen-2 rats+STZ (55 mg/kg body weight)	BG↑	albumin excretion rate↑	glomerulosclerosis; tubulointerstitial fibrosis	TRX—TXNIP↑	Sprague–Dawley rat mesangial cells; proximal tubule; distal tubule/collecting duct cells	increased expression of TXNIP	High glucose leads to an impairment of the Trx system, as a consequence of multiple changes that include increased expression of the endogenous TRX inhibitor, TXNIP, and reduced TRX activity.	(9)
			HbA1c↑							
2011	Sih Min Tan	heterozygous Ren-2 rats+STZ (55 mg/kg body weight)	BG↑	albumin excretion rate↑	increased tubulointerstitial matrix	TXNIP↑	NA	NA	Tranilast attenuates the up-regulation of TXNIP and oxidative stress in DKD.	(11)
2014	Chunling Huang	C57BL/6 J mice+STZ (55 mg/kg body weight)	BG↑			TXNIP ↑	HK-2 cells	NA	Tubular autophagic flux is disrupted in DKD an links the overexpression of TXNIP to the dysregulation of tubular autophagy.	(15)
						LC3/LC3-II↑ p62↑				
2015	Anu Shah	C57BL/6 mice+STZ (40 mg/kg body weight)	BG↑	SCR↑	interstitial fibrosis; glomerular extracellular matrix expansion; reduction in podocyte number; increased glomerular basement membrane thickness	ROS/reactive nitrogen species↑	Immortalized human podocytes	NA	A lack of TXNIP protects against DKD and upregulation of TXNIP by HG is a key mediator of early oxidative stress and a trigger for the development and progression of DKD.	(16)
			HbA1c↑	Cystatin C↑		Nox4↑				
			Insulin↓	24 h albuminuria↑ UACR↑		IL-1β↑					24 h urine protein↑	TNF-α↑
						Caspase3↑				
				2016		Ferhan S. Siddiqi					SD rats+STZ (60 mg/kg body weight)	BG↑	24 h urine protein↑		TXNIP↑	immortalized mouse podocytes	NA	EZH2 represses the transcription factor Pax6, which controls expression of the antioxidant inhibitor TXNIP, and in diabetes, downregulation of EZH2 promotes oxidative stress.	(12)
HbA1c↑																			
2016	Yang De Marinis	Sur1-E1506K^+/+^ mice	BG↑		increased mesangial matrix and mesangial cells	TXNIP gene↑	mouse SV40 MES13 mesangial cells; human mesangial cell line	NA	Glucose-stimulated TXNIP gene expression can be reversed by inhibition of HAT, or enhanced by inhibition of HDAC.	(13)
2016	Chunling Huang	mRen-2 rats STZ (55 mg/kg body weight)	BG↑		increased renal interstitial collagen deposition	TXNIP↑	HK-2 cells	increased expression of LC3 and P62 in the renal tubule cells	TXNIP inhibition suppressed diabetes induced autophagy and activation of the mTOR signaling pathway.	(17)
						LC3↑				
						P62↑				
						BNIP3↑				
						p-mTOR↑				
						p70S6↑				
2016	Chunyang Du	C57BL/6 J mice+STZ (50 mg/kg body weight)	BG↑	SCR↑	enhanced lipid droplets accumulation in the tubular cells	TXNIP↑	HK-2 cells	NA	TXNIP deficiency alleviates diabetic renal lipid accumulation by inhibiting fatty acid synthesis and promoting fatty acid-oxidation, which is maybe partly through the inhibition of Akt/mTOR signaling pathway activation.	(18)
			TG↑	BUN↑		SREBP-1↑				
				UACR↑		FASN↑				
						ACC↑				
						PPARα↓				
						ACOX1↓				
						CPT1↓				
						p-mTOR↑				
						p-Akt↑				
2016	Yara A. Samra	SD rats+STZ (50 mg/kg body weight)	BG↑	SCR↑	swelling and hypercellularity of glomerular tufts; mild glomerular capillary basement membrane thickening; vacuolar changes in the tubular cytoplasm	pERK↑	NA	NA	The antioxidant and anti-inflammatory effects of cepharanthine and piperine which were accompanied by inhibition of NF-κB and NLRP3 activation might be helpful mechanisms to halt the progression of DKD.	(14)
				BUN↑		p-p38MAPK↑				
				UACR↑		p-JNK↑				
						TNF-α↑				
						IL1-β↑				
						TXNIP↑				
						NLRP3↑				
2017	Salwa M. K. Almomen	Obese ZSF1 rats	BG↑	BUN↑	glomerular atrophy; mesangial expansion; tubular dilation and atrophy	*Txnip gene*↑	Heat-sensitive mouse podocyte cell line	NA	Daily intake of whole grape powder reduces the progression of kidney disease in obese diabetic rats.	(10)
				UACR↑						
				24 h urine protein↑						
2018	Yachun Han	db/db mice	BG↑	BUN↑	glomerular mesangial matrix proliferation; basement membrane thickening; foot process fusion tubulo-interstitial fibrosis	TRX↓	HK-2 cells	mesangial expansion; tubular atrophy; elevated ROS levels; higher TXNIP, IL-1β, IL-18 and NLRP3 expression but lower TRX expression	Activation of the mtROS-TXNIP/NLRP3/IL-1β biological axis plays an essential role in kidney tubular injury in DKD; MitoQ attenuated the signaling pathway activation and thus alleviated renal cell apoptosis and fibrosis.	(19)
			TC↑	SCR↑		TXNIP↑				
			TG↑			NLRP3↑				
						Caspase-1↑				
						IL-1β↑				
2019	Linlin Ji	C57BL/6 J mice+HFD/STZ (130 mg/kg body weight)	BG↑	SCR↑	atrophy and apoptosis of proximal tubular cells; thickening of the tubular basement membrane; increased interstitial collagen fibers	TRX↓	HK-2 cells	renal proximal tubular cell injury	FoxO1 bound to the TXNIP and TRX promoter and regulates the oxidative stress balance maintained by TXNIP-TRX.	(20)
				UACR↑		TXNIP↑				
	
2019	Shan Song	C57BL/6 J mice+STZ (50 mg/kg body weight)	BG↑	SCR↑	phenotypic alterations of podocytes; reduction in podocyte number	Nox1 ↑	immortalized murine podocytes	increased expression of TXNIP, Nox1, and Nox4; mTOR activation	TXNIP could ameliorate phenotypic alterations of podocytes *via* inhibition of mTOR in DKD.	(21)
			TG↑	BUN↑		Nox4↑				
				24 h urine protein↑		Raptor↑				
						p-S6↑				
						p-AKTSER473↑				
2020	Xin An	C57BL/6 J mice+HFD/STZ (100 mg/kg body weight)	BG↑	BUN↑	mesangial matrix hyperplasia; renal vesicles; glomerular basement membrane thickening	NOX4	NA	NA	TXNIP/NLRP3 axis is an important pathway that regulates DKD induced by pyroptosis; Punicalagin protects against DKD by inhibiting TXNIP/NLRP3 axis.	(22)
				SCR↑		TRX↓				
				UACR↑		TXNIP↑				
						NLRP3↑				
						Caspase-1↑				
						IL-1β↑				
2021	Hong Sun	db/db mice	BG↑	SCR↑	enhanced lipid droplets accumulation in the tubular cells	TXNIP↑	HK-2 cells	NA	TXNIP knockdown mitigated the accumulation of renal tubular lipids in diabetes through the regulation of SCAP, thereby inhibiting the SCAP-SREBP-2 signaling pathway, resulting in reduced cholesterol uptake and synthesis.	(23)
			TC↑	BUN↑		SCAP↑ SREBP-2↑				
			TG↑	u-NGAL↑		HMGCoAR↑				
				β2-MG↑		LDLr↑				
				24 h urine protein↑						
2021	Nada H. Eisa	SD rats+STZ (50 mg/kg body weight)	BG↑		dilation in medullary tubules; mild interstitial edema fibrosis	AGE↑	NA	NA	Phenethyl isothiocyanate attenuated DKD progression in a dose dependent manner mainly *via* interruption of AGE/RAGE and NLPR3/TXNIP/NrF2 crosstalk.	(24)
						RAGE↑				
						Nrf2↓				
						TXNIP ↑				
						NLRP3↑				
						caspase-1↑				
						IL-1β ↑				
						IL-6↑				
						TNF-α↑				

STZ, streptozocin; BG, blood glucose; HbA1c, glycosylated hemoglobin A1c; TXNIP, thioredoxin-interacting protein; TRX, thioredoxin; LC3, microtubule-associated protein 1A/1B light chain 3B; DKD, diabetic kidney disease; SCR, serum creatinine; ROS, reactive oxygen species; Nox4, NADPH oxidase 4; IL-1β, interleukin-1β; TNF-α, tumor necrosis factor-α; HG, high glucose; EZH2, enhancer of zeste homolog 2; Pax6, paired box 6; HAT, histone acetyltransferase; HDAC, histone deacetylase; BNIP3, BCL2 and adenovirus E1B 19-kDa-interacting protein 3; p-mTOR, phosphorylated mammalian target of rapamycin; p70S6, protein S6 kinase, 70 kDa; TG, total triglyceride; BUN, blood urea nitrogen; UACR, urinary albumin creatinine ratio; SREBP, sterol regulatory element-binding protein; FASN, fatty acid synthase; ACC acetyl-CoA carboxylase; PPARα, peroxisome proliferatoractivated receptor α; ACOX1, acyl-coenzyme A oxidase 1; HK-2, human renal proximal tubular epithelial cells; CPT1, carnitine palmitoyltransferase 1; p-ERK, phosphorylated extracellular signal-regulated kinase; p-JNK, phosphorylated c-Jun N-terminal kinase; NF-κB, nuclear factor κB; Nox1, NADPH oxidase 1; SCAP, SREBP cleavage activating protein; HMGCoAR, 3-hydroxy-3-methylglutaryl coenzyme A reductase; LDLr, low-density lipoprotein receptor; AGEs, advanced glycation end products; RAGE, receptor of AGEs; Nrf2, nuclear factor erythroid 2-related factor 2; IL-6, interleukin-6; NA, not available.

### General information on TXNIP

TXNIP, also referred to as Vitamin D3-upregulated protein 1 (VDUP-1), was first cloned and characterized in 1994 in HELA cells stimulated with Vitamin D3 (25). Subsequently, TXNIP was identified as a thioredoxin (TRX)-binding protein using the yeast two-hybrid system, which can suppress the expression and activity of TRX, and thus, it was called thioredoxin-binding protein-2 (TBP-2) (26). Studies on the TXNIP promoter could not identify the consensus vitamin-D3 response element. Moreover, vitamin-D3-mediated TXNIP transcription was not verified in other cells (27). A nonsense mutation in the TBP-2 gene was discovered in another study on HcB-19 mice, causing the mice to develop familial combined hyperlipidemia; thus, VDUP1/TBP-2 was called TXNIP (28). The human TXNIP gene is located on chromosome 1q21.1 and has eight introns and eight exons that cover 4,174 bp. Its nucleotide sequences are highly homologous to those of mouse (89%) and zebrafish (77%), suggesting that the functions of TXNIP are essential (25). The human TXNIP protein is a member of the α-arrestin protein family and contains 391 amino acids with a molecular weight (MW) of 46 kDa. TXNIP has an arrestin-like C-terminus (175–298 aa) as well as an arrestin-like N-terminus (10–152 aa) (29). There are two intramolecular disulfide bonds between Cys-247 and Cys-63 in TXNIP, which may help TXNIP to efficiently interact with TRX and inhibit its activity (26).

### TXNIP expression in the kidney of diabetic patients

In 2009, Andrew Advani first reported TXNIP in normal humans *via in situ* hybridization. TXNIP was expressed in the renal arteriole endothelium, glomeruli, collecting ducts, and distal convoluted tubules. Additionally, the researchers conducted qRT-PCR using renal biopsy samples. They extracted the mRNA from DKD cases, as well as from healthy renal tissues collected from the contralateral side of tumor cases receiving nephrectomy. Their results suggested that TXNIP was upregulated in patients with DKD (9). In 2018, Yachun Han found that TXNIP levels were significantly higher in the renal tissues of DKD patients compared to those of healthy controls by performing immunohistochemistry (IHC) and semi-quantification (19). In another study, IHC analysis showed that TXNIP expression was significantly higher in the glomeruli of patients with DKD relative to its expression in the glomeruli of normal controls (21). Despite the fact that these studies consistently showed high renal expression of TXNIP in DKD patients, the number of cases in the studies was small. Therefore, a large number of kidney samples from DKD patients must be collected for further research. Furthermore, serum TXNIP levels in diabetics and DKD patients were significantly higher than in healthy volunteers (30). Although some studies on serum TXNIP levels in patients with diabetes have yielded consistent results (31, 32), there is insufficient evidence on whether it can be used as a serum marker in patients with diabetes or DKD. Furthermore, another study has revealed that TXNIP expression was higher in the urinary sediment in type 1 diabetics with DKD, which was related to a reduction in the estimated glomerular filtration rate (eGFR) levels (33). These findings suggest that TXNIP expression in serum, urine, and kidney is significantly higher in DKD patients and that TXNIP may play an important role in the progression of DKD. Therefore, more research into the signal transduction role of TXNIP in DKD progression is critical.

## TXNIP signaling in DKD

### ROS-TRX-TXNIP-NLRP3 pathway

Reactive oxygen species (ROS) are generated in mammalian cells to mediate different physiological responses, such as cell growth, invasion, and differentiation. Oxidative stress (OS) is a major pathophysiological mechanism linked to a variety of diseases, including diabetes and DKD. TXNIP is a key mediator of the high glucose (HG)-induced OS. TXNIP and TRX active site thiols can form mixed disulfide bonds, which inhibit TRX activity while increasing ROS production (34). Mitochondria is the main (90%) ROS source in cells (35). Additionally, an increase in the production of mitochondrial ROS (mtROS) in the renal tissues of DKD cases and diabetic model rodents had a strong effect on the occurrence of DKD (19). mtROS is the main factor that activates the NLRP3 inflammasome (36). Additionally, TXNIP is related to activating the NLRP3 inflammasome in DKD (19, 22, 24). When TXNIP is released from TRX, it can bind to NLRP3 *via* a leucine-rich repeat domain, activating the NLRP3 inflammasome in response to mtROS stimulation. The activated NLRP3 can then cleave interleukin-1β (IL-1β) and interleukin-18 (IL-18) precursors, inducing an immunoinflammatory response in the kidneys of diabetics. MitoQ (a mitochondria-targeted antioxidant)-induced reduction in mtROS expression in the renal tissues of db/db mice and HK-2 cells can mitigate the activation of the TXNIP-NLRP3 inflammasome pathway, thereby alleviating renal cell inflammation, fibrosis, and apoptosis (19). Besides being produced in the mitochondria, cellular ROS can also be produced *via* the NADPH oxidase (Nox) system in the diabetic state. There are three Nox isoforms identified within renal tissues, including Nox1, Nox2, and Nox4; the isoform(s) that can induce ROS during diabetes is undetermined (37). However, Nox4 downregulation was shown to suppress the dissociation of TXNIP from TRX and inhibit the NLRP3 inflammasome from being activated in a DKD mouse model constructed by administering a high-fat diet (HFD), as well as injecting streptozotocin (STZ) intraperitoneally (22). Therefore, mtROS and Nox-mediated ROS generation significantly affect the TXNIP-NLRP3 inflammasome axis. TXNIP can modulate HG-mediated TRX activity impairment, whereas silencing TXNIP can abolish HG-mediated collagen generation and OS in diabetic kidneys (9). In a recent study, Shah et al. demonstrated DKD resistance in diabetic TXNIP−/−mice, a reduction in ROS production, Nox4 level, inflammation, and tubulointerstitial fibrosis. The renal function was preserved compared to the levels of these factors/activities in diabetic TXNIP+/+ mice (16). Wang et al. obtained similar results in TXNIP knockout diabetic mice (38). These results indicated the important effect of TXNIP on intracellular ROS production and NLRP3 inflammasome activation, which play key roles in the process of DKD.

### FOXO1-TXNIP

Forkhead transcription factor O1 (FOXO1) belongs to the forkhead box-containing transcription factor O family. FOXO1 can mediate OS within endothelial cells, renal proximal tubular cells (RPTCs), and pancreatic β-cells along with TRX and TXNIP (20, 39). Linlin et al. discovered that by binding to the promoters of TRX and TXNIP, FOXO1 reduced albuminuria, ROS generation, interstitial fibrosis, and RPTC apoptosis in HFD and STZ-induced diabetic mice and HG-treated RPTCs. Furthermore, it modulated ROS levels by increasing TRX expression while decreasing TXNIP expression (20). Our previous studies found that the FOXO1-TXNIP-NLRP3 inflammasome pathway was activated in RPTCs through ATP stimulation, resulting in the secretion of IL-1β (40). Although in-depth studies of this intracellular signaling pathway in the DKD state are lacking, it has been demonstrated that FOXO1 can suppress NLRP3 inflammasome activation by reducing the TXNIP level in patients with diabetic atherosclerosis and diabetic liver (41, 42). There is increasing evidence that FOXO1 plays an important role in the pathogenesis of DKD through the regulation of autophagy, apoptosis, and other cellular processes, which is independent of TXNIP (43). Therefore, FOXO1 may be another potential target for preventing and treating DKD.

### TXNIP and autophagy

Autophagy is responsible for transporting cytoplasmic contents into lysosomes to maintain homeostasis and cell functions. Apoptosis changes can cause cell injury and even cell death. In the case of diabetes-related metabolic changes or hyperglycemia, injured organelles and proteins accumulate, which is linked to the development of DKD. Autophagy changes in RPTCs and podocytes during diabetes (44). Microtubule-associated protein 1A/1B light chain 3B (LC3) is an autophagy marker that shows specific localization in autophagic structures during autophagy between phagophore and lysosomal degeneration. The LC3-I/LC3-II ratio (LC3 conversion) can be easily determined because LC3-II content is tightly associated with the quantity of autophagosome (45). The degradation of p62 is another important marker for monitoring autophagic activity since p62 can directly bind to LC3 and is selectively degraded through autophagy (46). TXNIP is an autophagy regulator in diabetes. Downregulation of TXNIP restores LC3-II and p62 protein levels and reduces autophagic flux in the HG-exposed rat retinal Müller cells (rMC-1). Thus, TXNIP downregulation can improve the visual function of diabetic rats (47). Chunling et al. were the first to report the upregulation of p62, TXNIP, and LC3/LC3-II in DKD mouse RPTCs and the formation of autophagic vacuoles in human RPTCs treated with HG. TXNIP silencing reduced p62 and LC3-II levels, as well as autophagic vacuoles, in HG-treated human RPTCs (15). Later, Chunling et al. also showed the dysfunction of tubular autophagy in renal tissues of DKD rats and patients. Their results suggested that the abnormal p62 and LC3 levels were normalized in the renal tissues of the TXNIP DNAzyme-treated diabetic rats. Additionally, HG dysregulated tubular mitophagy in RPTCs *in vitro*, and the effect was reversed by TXNIP siRNA, which inhibited mammalian target of rapamycin (mTOR) (17), an important factor related to the key regulatory mechanisms of autophagy. Based on the above studies, we hypothesized that balance autophagy by regulating TXNIP might be a prospective treatment for DKD in clinical.

### TXNIP and lipid

Lipometabolic disorder can cause ectopic renal lipid deposition in individuals with diabetes. Renal lipid concentrations, measured by MRI, are considerably higher in diabetics compared to that non-diabetic subjects (48). Furthermore, based on tissue staining results, the aberrant lipid droplets deposited in the interstitium, mesangial cells, and renal glomeruli suggest impairment of renal morphology and function, particularly in the renal tubules of diabetics (49, 50). Recent studies have suggested the key effect of TXNIP on lipid metabolism during diabetes (51). Chunyang et al. reported that TXNIP knockdown suppressed fatty acid (FA) production and promoted FA oxidation to mitigate lipid accumulation in HG-treated RPTCs and renal tissues of STZ-induced diabetic mice. This was partially achieved due to the activation of the mTOR pathway (18). Additionally, we found TXNIP knockdown can suppress cholesterol synthesis and uptake to mitigate lipid accumulation in the RPTCs of db/db mice, thus, improving the morphology and function of renal tubules (23). Though there have been few studies on the effects of TXNIP on diabetic renal lipid metabolism, it is critical to further investigate the lipid regulatory function of TXNIP. In the future, TXNIP could be used to treat renal lipid disorders.

## Conclusion and future perspective

**Figure 1:**
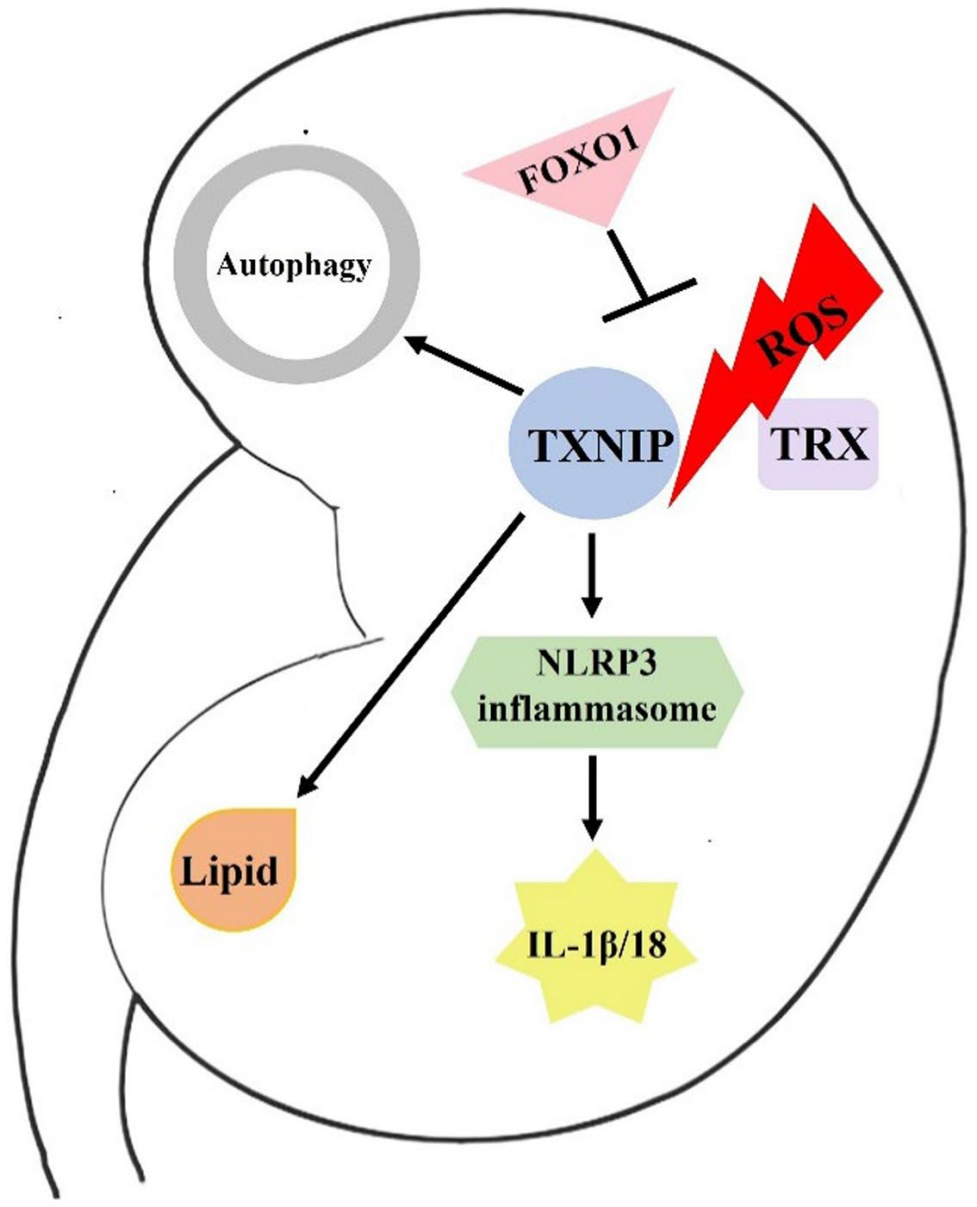
Brief mechanism of TXNIP in DKD. In response to ROS stimulation, TXNIP is released from TRX. Then, it binds to NLRP3 and activates the NLRP3 inflammasome. The activated NLRP3 can then cleave IL-1β and IL-18 precursors, inducing an immunoinflammatory response in the kidneys of diabetics. FOXO1 may ameliorate DKD by affecting ROS generation and TXNIP-TRX pathway. In addition, TXNIP also damages renal cell autophagy and lipid metabolism, resulting in the development of DKD.

Over the years, significant advances have been made in TXNIP biology and its role in metabolic regulation. As mentioned above, TXNIP plays a role in renal oxidative stress, inflammation, autophagy, and lipid metabolism during DKD and may be a key regulatory factor for the pathogenic mechanism and development of DKD (Figure 1). However, more research is needed to fully understand the mechanism underlying TXNIP’s physiological activities in DKD, and targeting TXNIP may offer a unique treatment strategy to combat the global DKD problem.

## Author contributions

HS designed the article and wrote the first draft. RS and YH searched the literature. XS and QL revised the manuscript. All authors read and approved the final manuscript.

## Funding

This work was supported by the National Natural Science Foundation of China (grant no. 82270755 to HS), Suzhou Science and Technology Project (grant no. SYS2020104, SZM2022013 to HS and SZM2021013 to XS).

## Conflict of interest

The authors declare that the research was conducted in the absence of any commercial or financial relationships that could be construed as a potential conflict of interest.

## Publisher’s note

All claims expressed in this article are solely those of the authors and do not necessarily represent those of their affiliated organizations, or those of the publisher, the editors and the reviewers. Any product that may be evaluated in this article, or claim that may be made by its manufacturer, is not guaranteed or endorsed by the publisher.
